# Concurrent TB and HIV therapies effectively control clinical reactivation of TB during co-infection but fail to eliminate chronic immune activation

**DOI:** 10.21203/rs.3.rs-4908400/v1

**Published:** 2024-08-26

**Authors:** Riti Sharan, Yi Zou, Zhao Lai, Bindu Singh, Vinay Shivanna, Edward Dick, Shannan Hall-Ursone, Shabaana Khader, Smriti Mehra, Xavier Alvarez, Jyothi Rengarajan, Deepak Kaushal

**Affiliations:** Texas Biomedical Research Institute; UTHSCSA; The University of Texas Health San Antonio; Texas Biomedical Research Institute; Texas Biomedical Research Institute; Southwest National Primate Research Center; Texas Biomedical Research Institute; University of Chicago; Texas Biomedical Research Institute; Texas Biomedical Research Institute; Emory University; Southwest National Primate Research Center, Texas Biomedical Research Institute

**Keywords:** cART, 3HP, TB/SIV co-infection, LTBI, reactivation

## Abstract

The majority of Human Immunodeficiency Virus (HIV) negative individuals exposed to *Mycobacterium tuberculosis* (*Mtb*) control the bacillary infection as latent TB infection (LTBI). Co-infection with HIV, however, drastically increases the risk to progression to tuberculosis (TB) disease. TB is therefore the leading cause of death in people living with HIV (PLWH) globally. Combinatorial antiretroviral therapy (cART) is the cornerstone of HIV care in humans and reduces the risk of reactivation of LTBI. However, the immune control of *Mtb* infection is not fully restored by cART as indicated by higher incidence of TB in PLWH despite cART. In the macaque model of co-infection, skewed pulmonary CD4^+^ T_EM_ responses persist, and new TB lesions form despite cART treatment. We hypothesized that regimens that concurrently administer anti-TB therapy and cART would significantly reduce TB in co-infected macaques than cART alone, resulting in superior bacterial control, mitigation of persistent inflammation and lasting protective immunity. We studied components of TB immunity that remain impaired after cART in the lung compartment, versus those that are restored by concurrent 3 months of once weekly isoniazid and rifapentine (3HP) and cART in the rhesus macaque (RM) model of LTBI and Simian Immunodeficiency Virus (SIV) co-infection. Concurrent administration of cART + 3HP did improve clinical and microbiological attributes of *Mtb*/SIV co-infection compared to cART-naïve or -untreated RMs. While RMs in the cART + 3HP group exhibited significantly lower granuloma volumes after treatment, they, however, continued to harbor caseous granulomas with increased FDG uptake. cART only partially restores the constitution of CD4 + T cells to the lung compartment in co-infected macaques. Concurrent therapy did not further enhance the frequency of reconstituted CD4^+^ T cells in BAL and lung of *Mtb*/SIV co-infected RMs compared to cART, and treated animals continued to display incomplete reconstitution to the lung. Furthermore, the reconstituted CD4^+^ T cells in BAL and lung of cART + 3HP treated RMs exhibited an increased frequencies of activated, exhausted and inflamed phenotype compared to LTBI RMs. cART + 3HP failed to restore the effector memory CD4^+^ T cell population that was significantly reduced in pulmonary compartment post SIV co-infection. Concurrent therapy was associated with the induction of Type I IFN transcriptional signatures and led to increased *Mtb*-specific T_H1_/T_H17_ responses correlated with protection, but decreased *Mtb*-specific TNFa responses, which could have a detrimental impact on long term protection. Our results suggest the mechanisms by which *Mtb*/HIV co-infected individuals remain at risk for progression due to subsequent infections or reactivation due of persisting defects in pulmonary T cell responses. By identifying lung-specific immune components in this model, it is possible to pinpoint the pathways that can be targeted for host-directed adjunctive therapies for TB/HIV co-infection.

## Introduction

Combinatorial antiretroviral therapy (cART) reduces the risk of reactivation of latent tuberculosis infection (LTBI) in humans [1–4]. However, tuberculosis (TB) remains a major cause of morbidity and mortality in people living with HIV (PLWH) [5–7]. Anti-tubercular treatment has been shown to reduce disease incidence by 30–50%[8, 9] and is recommended for PLWH in high-burden countries. Observational studies in humans suggest that concurrent administration of cART and Isoniazid Preventive Therapy (IPT) for LTBI lowers the risk of developing TB compared to cART alone [10]. A randomized double-blind placebo-controlled trial [8] showed that administering IPT in conjunction with cART resulted in significantly lower numbers of incident TB cases than cART plus placebo. Thus, concurrent cART + IPT leads to improved outcomes with clear protective effects and clinical benefit to HIV-infected individuals. Although the WHO recommends concurrent IPT and cART in TB-endemic settings, uptake remains poor and the immune mechanisms underlying the benefits of concurrent cART and IPT have not been defined. Another caveat of this approach has been a lack of completion of treatment regimen by majority of those who initiate the 6-month course of daily isoniazid while on cART. To enable treatment adherence and completion, the World Health Organization (WHO) recommended 12-dose weekly regimen of Isoniazid and Rifapentine (3HP) as a treatment-shortened option for treating TB. Concurrent administration of cART and 3HP was safe and well tolerated with over 95% completion rate [11, 12]. In our RM model of *Mycobacterium tuberculosis* (*Mtb*)/SIV co-infection, though 3HP effectively reduced persistent LTBI [12], it did not sterilize the lungs of a third of the treated RMs [13]. Recent work in *Mtb*/SIV co-infected RMs [14] shows that in addition to the depletion of CD4^+^ T cells, HIV-driven chronic immune activation correlates with LTBI reactivation [14–18]. Though cART controls viral replication, it leads to insufficient reconstitution of protective CD4^+^ T effector memory (T_EM_) responses in the lungs [15] and fails to rescue from virus-driven immune activation [19]. Administration of anti-tubercular therapy, concurrently with cART reduces reactivation significantly better than cART among individuals with LTBI [11]. However, long-term sterilization of bacteria and immune reconstitution in the lungs has not been shown in these individuals.

In this study, we leveraged our nonhuman primate (NHP) model of *Mtb*/SIV co-infection to study effect of simultaneous cART and 3HP treatment on immune responses to *Mtb*. Performing these studies in humans is virtually impossible due to lack of determination of timing of *Mtb* and HIV co-infections, verification of bacterial and viral loads and performance of invasive longitudinal studies to investigate the lung compartment. Our very low-dose aerosol infection NHP model recapitulates the spectrum of human lung pathological lesions, including LTBI and its reactivation to active TB by HIV [13–15, 17, 20]. We carried out detailed studies of the immune responses by longitudinal sampling of blood and bronchoalveolar lavage (BAL) in the absence of cART as well as during and after cART and cART + 3HP. Importantly, we investigated the functional capacity of antigen-specific T cell immunity in the lung microenvironment. Our findings clearly show that while cART and 3HP control viral and bacterial replication, respectively, there is partial immune reconstitution and with the reconstituted CD4^+^ T cell exhibited highly impaired functional capabilities. In particular, skewed CD4^+^ T effector memory responses persist despite concurrent cART and anti-TB treatment in *Mtb*/SIV co-infected macaques and the ongoing inflammation in the lung is not ameliorated. It is well known that PLHIV and TB remain at risk for progression due to subsequent infections or TB reactivation even after improved clinical and microbiological attributes. We conclude that persisting immune activation/inflammation are the mechanisms that cause this susceptibility. Clearly host directed therapies against immune activation and lung inflammation, adjunctive to TB therapy and cART must be developed to better treat PLHIV with TB.

## Results

### Concurrent treatment with cART and 3HP improves clinical and microbiological attributes of Mtb/SIV co-infection.

**Figure 1:**
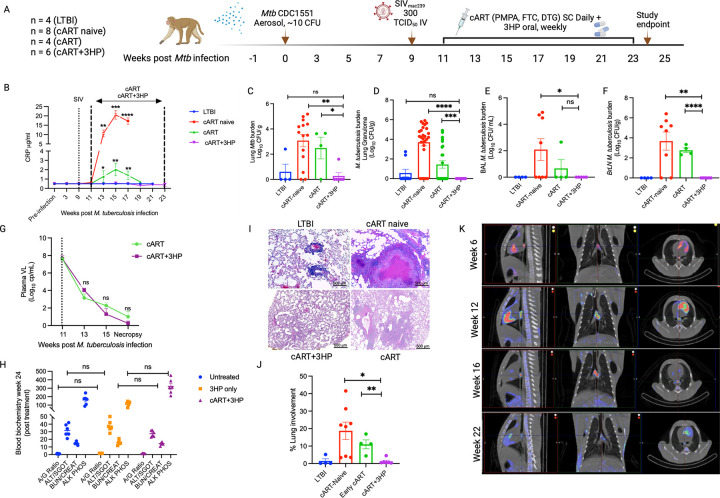
Concurrent treatment with cART and 3HP improves clinical and microbiological attributes of *Mtb*/SIV co-infection. (A) Study outline. (B) Serum CRP levels. Bacterial burden (log_10_CFU/g or log_10_CFU/mL) was determined in (C) lung, (D) lung granulomas, (E) bronchoalveolar lavage (BAL), (F) bronchial lymph nodes (BrLN) at necropsy by homogenizing the tissues and plating on agar plates. (G) Viral loads in plasma of cART and cART+3HP-treated RMs were measured longitudinally throughout the study. (H) Blood biochemistry for serum albumin/globulin (A/G) (g/dL) ratio, aspartate aminotransferase or serum glutamic-oxaloacetic transaminase (ALT/SGOT) (units per liter of serum), blood urea nitrogen/creatinine (BUN/creat) (μmol/L) ratio, and alkaline phosphatase (Alk phos) (units per liter), at week 24 after TB infection or 1-week after treatment completion for both cART and cART+3HP-treated groups. (I) To determine the impact of cART+3HP on lung pathology, lung tissue was collected at necropsy and stained with H&E to study the cellular and granulomatous pathology in LTBI (*n* = 4), cART naive (*n* = 8), cART (*n* = 4), and cART+3HP (*n* = 6). Scale bars: 500μm. (J) Percentage of lung involvement was calculated by a board-certified pathologist by quantification of the number of lesions per lobe. (K) Representative PET scans of cART+3HP treated RM at week 6 (LTBI), week 12 (LTBI+SIV), week 16 (LTBI+SIV+cART+3HP) and week 22 (end of cART+3HP treatment). Significance was determined in LTBI (*n* = 4), cART naive (*n* = 8), cART (*n* = 4), and cART+3HP (*n* = 6) using 1-way ANOVA with Tukey’s multiple-comparison test. *P < 0.05; **P < 0.01; ***P < 0.001; ****P < 0.0001. Data are presented as mean ± SEM.

To assess the impact of concurrent cART and 3HP therapy on LTBI reactivation in *Mtb*/SIV co-infection, we utilized 6 new RMs and reused published data from LTBI (*n* = 4), cART -naïve coinfected RMs (*n* = 8) and co-infected RMs treated with cART alone for 9 weeks (*n* = 4) (refs 15, 19 and Supplemental Table 2). The study design is outlined in Fig. 1A. All the RMs were infected with a low dose of *Mtb* (~ 10 CFU deposited in the lungs) and subsequently with SIV (300 TCID_50_ SIV_mac239_, intravenous). Infection was confirmed by a positive tuberculin skin test at weeks 3 and 5 after *Mtb* infection. All study RMs developed LTBI infection characterized by less than 1 to 2 Log_10_CFU of *Mtb* in the bronchoalveolar lavage (BAL) at weeks 3, 5 and 7 post *Mtb* infection, serum C-reactive protein (CRP) of 5 μg/mL or lower (Fig. 1B), and no significant change in percentage body temperature (Supplemental Fig. 1A) and body weight (Supplemental Fig. 1B) up to 9 weeks after *Mtb* infection. Upon establishment of latency, RMs were coinfected with 300 TCID_50_ SIV_mac239_ via the intravenous route 9 weeks after *Mtb* infection [14, 15, 20]. SIV infection was confirmed by measuring the plasma viral loads via reverse transcriptase quantitative PCR (RT-qPCR). The RMs were either treated with cART alone or cART + 3HP, once weekly orally for 12 weeks (Fig. 1A) and euthanized at treatment completion. Clinical, pathological and immunological response was compared in the 4 experimental groups: LTBI, cART naïve, cART and cART + 3HP.

The RMs in cART + 3HP group survived in good body condition with adequate body muscling and fat until the predetermined study endpoint. RMs in cART naïve group were humanely euthanized on prespecified endpoints starting as early as 2 weeks post SIV co-infection (Supplemental Fig. 1C). Elevated serum CRP levels associate with active TB and increase in bacterial burdens in NHPs [15, 17, 20]. CRP levels in cART + 3HP were significantly lower than cART naïve (*P < 0.0001*) and cART treated RMs (*P < 0.001*) (Fig. 1B). More importantly, the CRP levels in cART + 3HP RMs was not significantly different from LTBI RMs (*P* = 0.44). To determine the impact of cART + 3HP on bacterial burden, BAL fluid, lungs, bronchial lymph nodes and lung granulomas were plated on 7H11 agar plates as previously described [14, 15, 17, 20]. 5 out of 6 RMs in cART + 3HP group had no detectable bacterial burden in lung collected at necropsy, compared to just 1 out of four cART-treated and 2 out of 14 cART-naive RMs and both differences were statistically significant (Fig. 1C). Thus, the cART + 3HP group behaved comparable to the LTBI (SIV uninfected) group with 87.5% and 75% of the lung samples being sterile respectively in these groups. All 6 RMs in cART + 3HP group were devoid of detectable bacilli in lung granulomas (Fig. 1D), BAL (Fig. 1E) and bronchial lymph nodes (Fig. 1F) at necropsy. Additionally, the bacterial burden in cART + 3HP RMs was significantly lower than cART treated RMs in lung (*P = 0.01*), lung granulomas (*P = 0.001*) and bronchial lymph node (*P < 0.0001*). In contrast 81% RMs harbored bacilli in lung granulomas and 100% of study animals had detectable bacilli in bronchial lymph nodes when treated with cART alone (Fig. 1D and 1F).

To evaluate the efficacy of cART regimen in presence of 3HP treatment, viral loads were measured in the plasma of all 6 RMs and compared with cART treated RMs at pre-determined time points post cART initiation (Fig. 1G). There was no significant difference in the rates of decay of the viral loads in either group. Thus, 3HP did not alter the efficacy of cART in controlling viral replication. We also studied cytotoxicity markers in blood to determine safety of administering cART + 3HP relative to untreated and 3HP treated cohorts from archived samples. We did not observe any significant change in the levels of serum albumin/globulin (A/G) (g/dL) ratio, aspartate aminotransferase or serum glutamic-oxaloacetic transaminase (ALT/SGOT) (units per liter of serum), blood urea nitrogen/creatinine (BUN/creat) (μmol/L) ratio, and alkaline phosphatase (Alk phos) (units per liter), at week 24 after TB infection or 1 – week after treatment completion (Fig. 1H) in untreated, 3HP treated and cART + 3HP treated RMs. To determine the impact of cART + 3HP treatment on the lung cellular and granulomatous pathology, lung tissue sections collected at necropsy were stained with hematoxylin and eosin (H&E) (Fig. 1I) and findings analyzed by board-certified (Dipl, American College of Veterinary Pathologists) pathologists. The pathological findings correlated with the clinical and microbiological observations. There were a few, scattered, non-necrotizing and caseous granulomas in the lung lobes of approximately 0.5–1 cm in size in cART + 3HP treated RMs. There were rare, small aggregates of lymphocytes and macrophages in some lung sections. A single RM demonstrated multifocal accumulations of lymphocytes and non-necrotizing active granulomas in the liver. Overall, hilar, bronchial lymph nodes, spleen and other tissues were observed to have normal pathology comparable to LTBI only controls. cART + 3HP RMs demonstrated significant decrease in percentage lung involvement in pathology compared to cART and cART naïve RMs (Fig. 1J). Overall, lung of cART + 3HP treated RMs harbored less lesions compared to cART-naïve RMs. Lung of cART naïve and cART alone treated RMs showed numerous large granulomas with necrotic cores. Thus, administration of cART + 3HP is safe, efficacious in controlling bacterial burden and improved pathology compared to cART treated RMs.

We performed Positron Emission Tomography with Computed Tomography (PET/CT) to study lung lesions in 3 of the 6 RMs at weeks 6 (LTBI), 12 (LTBI + SIV co-infection, one week post cART + 3HP initiation), 16 (4 weeks post cART + 3HP initiation) and 22 (10 weeks post cART + 3HP initiation) (Fig. 1K). The lung lesions in all RMs remained stable, i.e., no or minimal progression in size and architecture at week 6 after infection, confirming LTBI (Fig. 1K). All three RMs that were scanned showed significant increase (*P* = 0.01) 18F-fluorodeoxyglucose (18F-FDG) uptake in lung upon SIV co-infection and 1 week of cART + 3HP treatment at week 12 post *Mtb* infection indicating progression of TB pathology (Supplementary Fig. 1E). Scans at week 16 post *Mtb* infection (4 weeks of cART + 3HP treatment) showed decreased 18F-FDG uptake, though the decrease was not significant. We did not observe a further increase in volume of lung lesions (Supplementary Fig. 1D) or uptake of 18F-FDG (Supplementary Fig. 1E) at week 22 post *Mtb* infection (10 weeks of cART + 3HP treatment). PET/CT results therefore demonstrate a significant decrease in volume of lesions but not in their metabolic potential post cART + 3HP treatment, suggesting that concurrent treatment led to a progressively increased resolution of caseous lesions that had been formed post SIV co-infection (week 12) but did not reduce the ongoing inflammation in the few remaining lesions.

### Immune reconstitution by cART + 3HP in pulmonary compartment of Mtb/SIV co-infected RMs.

**Figure 2:**
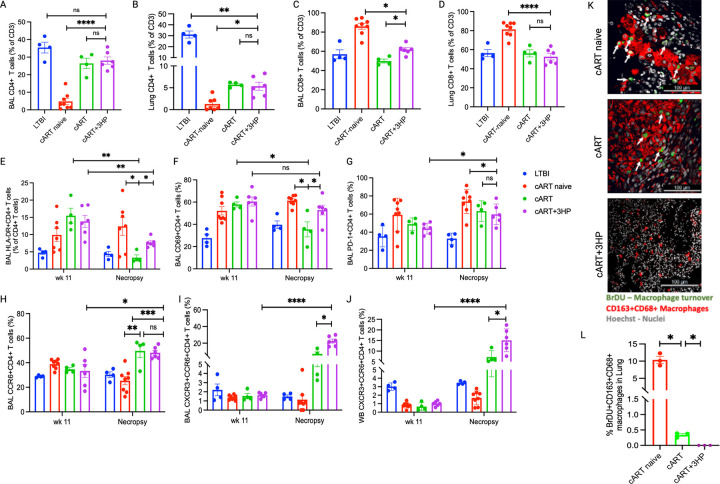
Treatment of *Mtb*/SIV co-infected RMs with cART+3HP increases migration of T_H17_ and T_H1*_ cells into pulmonary compartment compared to cART naïve RMs. We measured percentages of CD4^+^ T cells in (A) BAL and (B) lung, and percentages of CD8^+^ T cells in (C) BAL and (D) in LTBI (*n* = 4), cART naive (*n* = 8), cART (*n* = 4), and cART+3HP (*n* = 6) RMs to measure immune reconstitution in *Mtb*/SIV co-infected. To study impact of cART+3HP on immune activation, we measured the percentages of (E) BAL HLA-DR^+^CD4^+^ T cells, (F) BAL CD69^+^CD4^+^ T cells, and (G) PD-1^+^CD4^+^ T cells at peak viremia (week 11 post-*Mtb* infection or 2 weeks post SIV co-infection of LTBI) and at necropsy. We determined T_H17_ and T_H1*_ response in pulmonary and peripheral compartments by measuring percentage of (H) BAL CCR6^+^CD4^+^ T cells, (I) BAL CXCR3^+^CCR6^+^CD4^+^T cells and (J) WB CXCR3^+^CCR6^+^CD4^+^ T cells) at peak viremia (week 11 post-*Mtb* infection or 2 weeks post SIV co-infection of LTBI) and at necropsy. (K) We performed immunohistochemistry to study impact of cART+3HP treatment on macrophage turnover by staining for BrDU+ nuclei (green, indicated with white arrows) of macrophages (CD163+CD68+, red) per μm^2^ of lung sections of cART naïve, cART and cART+3HP treated RMs. (L) The images were analyzed using HALO software and captured on Axio Scan Z1. Significance was determined using 2-tailed Student’s t test. *P < 0.05.

Immunophenotyping of T cells was performed to assess both the extent and the quality of immune reconstitution by cART + 3HP relative to cART in pulmonary compartment of *Mtb*/SIV co-infected RMs. We have earlier demonstrated only partial restoration of depleted CD4^+^ T cells in BAL (Fig. 2A) and lung (Fig. 2B) after 12 weeks of cART in *Mtb*/SIV co-infected RMs, with significantly lower frequencies in lung tissue than those in the LTBI animals. 12 weeks of cART + 3HP treatment reconstituted CD4^+^ T cell frequency in BAL to comparable levels of LTBI (Fig. 2A) but not in lung, where the CD4^+^ T cell frequency remained significantly lower than LTBI control (Fig. 2B) (*P* = 0.0021). A significantly increased percentage of CD8^+^ T cells was observed in BAL (Fig. 2C) of cART + 3HP RMs compared to cART treated RMs (*P = 0.04*) but not in lung (Fig. 2D). The percentage of CD8^+^ T cells were not significantly different in lung of LTBI, cART and cART + 3HP treated, *Mtb*/SIV co-infected RMs. We have previously shown that chronic immune activation drives LTBI reactivation upon SIV co-infection in RMs [14, 15, 20]. To assess the impact of cART + 3HP on T cell activation, we studied expression of HLA-DR and CD69 on CD4^+^ T cells in BAL at week 11 post *Mtb* infection (or 2 weeks post SIV co-infection, prior to initiation of cART + 3HP) and at necropsy (end of 12 weeks of cART + 3HP treatment) in all 4 study groups. All *Mtb*/SIV co-infected groups exhibited increased frequencies of HLA-DR^+^- and CD69^+^- CD4^+^ T cells at week 11 (peak viremia) compared to the LTBI group (Fig. 2E, Fig. 2F). cART + 3HP effectively reduced the percentage of CD4^+^ T cells expressing HLA-DR and CD69 compared to cART naïve RMs, but not to the levels seen in LTBI or cART treated RMs. The increased activation of CD4^+^ T cells may be attributed to tuberculosis-immune reconstitution inflammatory syndrome (TB-IRIS) with concurrent cART + 3HP. High expression of PD-1 marker on T cells is often associated with increased exhaustion and T cell dysfunction in chronic infections such as HIV despite cART [21, 22]. To study the impact of cART + 3HP on T cell exhaustion in *Mtb*/SIV co-infection, we determined the percentage T cells expressing PD-1 in BAL cells at week 11 (peak viremia) and necropsy (Fig. 2G). cART and cART + 3HP treated RMs demonstrated significantly higher percentage of PD-1^+^CD4^+^ T cells compared to LTBI RMs at necropsy. Addition of 3HP to cART did not alleviate T cell exhaustion in pulmonary compartment as seen by no significant difference in PD-1 expressing CD4^+^ T cells in BAL between cART and cART + 3HP treated RMs (Fig. 2G). This was in spite the fact that virtually no detectable *Mtb* and SIV were present at the end of the protocol in the concurrently treated RMs. Overall, we conclude that cART + 3HP fails to control immune activation post SIV co-infection of LTBI leading to exhaustion of CD4^+^ T cells in pulmonary compartment. We hypothesize that the duration and magnitude of immune activation dictates the incapability of T cells to elaborate the usual array of functional effector responses in *Mtb*/SIV co-infection. It is important to note that increased turnover is not observed in the macrophages (Figs. 2K and 2L). A significantly lower (*P* < 0.05) percentage of macrophage turnover was observed in the lungs of RMs treated with cART + 3HP compared to cART and cART naïve RMs (Fig. 2L). A higher number of BrDU^+^ nuclei (green) within macrophages (red) as indicated by white arrows was seen in lung of cART naïve and cART treated RMs but was absent in lung of cART + 3HP treated RMs (Fig. 2K).

We further studied the impact of cART + 3HP on T_H17_ and T_H1*_ phenotypes in the pulmonary compartment of *Mtb*/SIV co-infected RMs. A significantly higher percentage of CD4^+^ T cells expressing CCR6, a regulator of migration and function of T_H17_ cells was observed in BAL cells of cART and cART + 3HP treated RMs (Fig. 2H) compared to LTBI and cART naïve RMs at necropsy. Similarly, we observed a significantly higher percentage of CD4^+^ T cells co-expressing CXCR3 and CCR6 in cART and cART + 3HP treated RMs compared to LTBI and cART naïve RMs, in both, BAL and peripheral blood cells (Figs. 2I and 2J). Additionally, cART + 3HP treated RMs harbored a significantly higher percentage of CXCR3^+^CCR6^+^CD4^+^ T cells (T_H1*_) in local and peripheral compartments compared to cART treated RMs (Figs. 2I and 2J). These findings align with our previous observation that higher frequencies of CD4^+^ T cells co-expressing CXCR3 and CCR6 associate with bacterial control in *Mtb*/SIV co-infection [23]. It has been previously reported that T_H1*_ subset is the most frequent *Mtb*-specific T cell subset in the lungs of latent TB donors and that their numbers are increased when compared to healthy subjects [24]. The higher percentage of CXCR3^+^CCR6^+^CD4^+^ T cells in local and peripheral compartments could also be attributed to cART mediated control of viral replication as CXCR3^+^CCR6^+^ cells are known to be preferential targets of HIV/SIV infection [24, 25]. Further, a reduction in this cell subset could be attributed to higher rates of LTBI reactivation. Thus, treatment of *Mtb*/SIV co-infected RMs with cART + 3HP increases migration of T_H17_ and T_H1*_ cells into pulmonary compartment compared to cART naïve RMs.

### Poor recovery of effector memory T cells by cART + 3HP in Mtb/SIV co-infected RMs.

**Figure 3:**
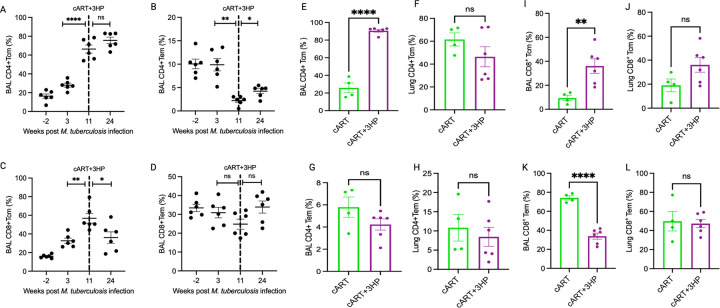
*Poor recovery of effector memory T cells in RMs treated with cART or cART+3HP*. We immunophenotyped the pulmonary CD4^+^ and CD8^+^ T cells in cART or cART+3HP treated RMs into CD^+^T_CM_ and CD4^+^T_EM_ populations. Percentage of (A) BAL CD4^+^ T_CM_, (B) BAL CD4^+^ T_EM_, (C) BAL CD8^+^ T_CM_ and (D) BAL CD8^+^ T_EM_ were measured in BAL of cART+3HP treated RMs at pre-infection, weeks 3, 11 and 24 post *Mtb*-infection. We compared the percentage of (E) BAL CD4^+^ T_CM_, (F) lung CD4^+^ T_CM_, (G) BAL CD4^+^ T_EM_, (H) lung CD4^+^ T_EM_, (I) BAL CD8^+^ T_CM_, (J) lung CD8^+^ T_CM_, (K) BAL CD8^+^ T_EM_, (L) lung CD8^+^ T_EM_ in cART versus cART+3HP treated RMs at necropsy. Significance was determined using 1-way ANOVA with Sidak’s or Tukey’s correction as applicable. *P < 0.05; **P < 0.01; ***P < 0.001; ****P < 0.0001. Data are presented as mean ± SEM.

To investigate functional immune reconstitution by cART + 3HP in pulmonary compartment of *Mtb*/SIV co-infected RMs, we further immunophenotyped the partially replenished CD4^+^ T cells into central memory (CD28^+^/CD95^+^) (CD4^+^T_CM_) and effector memory (CD28^−^/CD95^+^) T cells (CD4^+^T_EM_) (Supplementary Fig. 2). SIV co-infection of latent *Mtb* infection caused a significant increase in percentage of CD4^+^T_CM_ in BAL at week 11 (peak viremia prior to cART + 3HP treatment) (*P < 0.0001*) (Fig. 3A; Supplementary Fig. 3A). The increased percentage of CD4^+^T_CM_ persisted during and till end of the 12 week-long concurrent cART + 3HP treatment. On the contrary, a significant decline occurred in the frequency of CD4^+^T_EM_ in BAL at peak viremia which marginally increased at end of 12 weeks cART + 3HP treatment (Fig. 3B; Supplementary Fig. 3A). However, the percentage of CD4^+^T_EM_ at necropsy was significantly lesser than that seen in LTBI phase of the study (week 3 post *Mtb*-infection) (*P = 0.002*). These findings align with our previous observation that cART treatment fails to replenish the depleted CD4^+^T_EM_ in BAL and lung of *Mtb*/SIV co-infected RMs [15]. Immunophenotyping of BAL CD8^+^ T cells into CD8^+^T_CM_ and CD8^+^T_EM_ showed a significant increase (*P = 0.01*) in percentage of CD4^+^T_CM_ at peak viremia (week 11 post-*Mtb* infection or 2 weeks post SIV co-infection). This increase was mitigated by cART + 3HP as seen by marginally reduced percentage at necropsy (*P = 0.01*) (Fig. 3C; Supplementary Fig. 3B). No significant change was observed in percentage of CD8^+^T_EM_ in BAL at weeks 3, 11 and 24 (Fig. 3D; Supplementary Fig. 3B) (*P = 0.2*). Thus, cART + 3HP expands the CD4^+^ and CD8 + T_CM_ but is unable to replenish the CD4^+^T_EM_ in pulmonary compartment of *Mtb*/SIV co-infected RMs.

We further compared the restoration of CD4^+^T_CM_ and CD4^+^T_EM_ in BAL and lung of *Mtb*/SIV co-infected RMs treated with cART or cART + 3HP (Figs. 3E–3L). Despite similar percentage of CD4^+^ T cells in BAL at necropsy, there was a significantly higher percentage (*P < 0.0001*) of CD4^+^T_CM_ in cART + 3HP treated RMs compared to cART treated RMs (Fig. 3E). No significant difference was observed in lung CD4^+^T_CM_ (Fig. 3F), BAL CD4^+^T_EM_ (Fig. 3G) and lung CD4^+^ T_EM_ (Fig. 3H) between cART and cART + 3HP treated RMs. Similar to CD4^+^T_CM_, cART + 3HP RMs exhibited significantly higher (*P = 0.009*) percentage of CD8^+^T_CM_ in BAL (Fig. 3I) with a concurrent decrease in CD8^+^T_EM_ (P < 0.0001) (Fig. 3K) compared to cART treated RMs. However, there was no significant difference between lung CD8^+^T_CM_ (Fig. 3J) and CD8^+^T_EM_ (Fig. 3L) in cART and cART + 3HP treated RMs. Overall, there were dynamic changes in the memory phenotype of CD4^+^ and CD8^+^ T cells in BAL compared to lung in cART and cART + 3HP treated RMs. BAL is a critical resource to study longitudinal changes in pulmonary immune response and has been shown to be useful to evaluate local response to therapy [26, 27].

### cART + 3HP increases Mtb-specific T _H1_ /T _H17_ response in pulmonary compartment of Mtb/SIV co-infected RMs.

**Figure 4:**
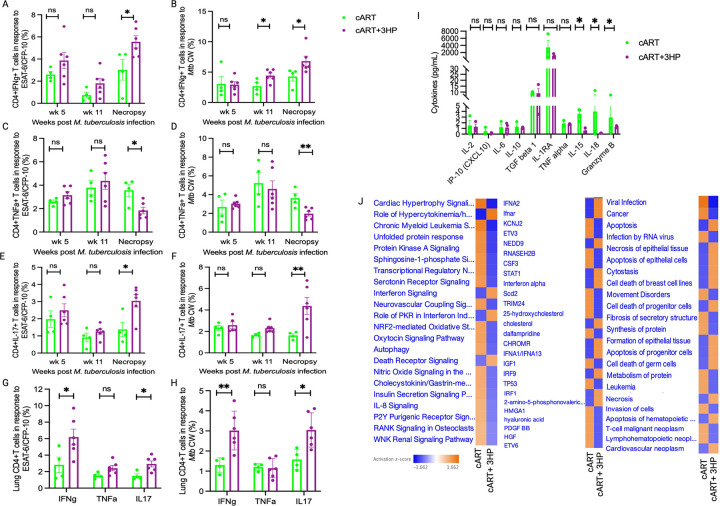
*cART+3HP increases Mtb-specific T*_*H1*_*/T*_*H17*_ *response in pulmonary compartment of Mtb/SIV co-infected RMs*. Percentage of CD4^+^IFNg^+^ T cells in response to (A) ESAT-6/CFP-10, (B) *Mtb* CW, CD4^+^TNFa^+^ T cells in response to (C) ESAT-6/CFP-10, (D) *Mtb* CW and CD4^+^IL-17^+^ T cells in response to (E) ESAT-6/CFP-10 and (F) *Mtb* CW were measured in BAL at weeks 5, 11 and necropsy in *Mtb*/SIV co-infected RMs treated with cART (*n* = 4) or cART+3HP (*n* = 6). Percentage of CD4^+^ T cells expressing either IFNg, TNFa or IL17 was measured in lung at necropsy in response to (G) ESAT=6/CFP-10 and (H) *Mtb* CW. Significance was determined using 1-way ANOVA with Sidak’s or Tukey’s correction as applicable. *P < 0.05; **P < 0.01; ***P < 0.001; ****P < 0.0001. Data are presented as mean ± SEM.

BAL samples were collected from study RMs at weeks 5, 11 and necropsy post *Mtb* infection using standard operating procedures by the veterinarian. Single cell suspensions were prepared as per the lab standardized protocol [28]. All *Mtb*-specific responses were background corrected (Supplementary Fig. 5). BAL cells were stimulated *ex vivo* with *Mtb*-specific antigens, ESAT-6/CFP-10 and *Mtb* Cell Wall Fraction (*Mtb* CW) for 16 h and stained with flow cytometry antibodies to detect IFNg, TNFa, and IL-17. A significantly higher percentage of IFNg expressing *Mtb*-specific CD4^+^ T cells was seen in BAL of cART + 3HP treated RMs at end of treatment when stimulated with ESAT-6/CFP-10 (Fig. 4A) (*P = 0.04*) and *Mtb* CW (Fig. 4B) (*P = 0.009*) compared to cART treated RMs. We hypothesize that cART + 3HP treatment effectively control bacteria thus enhancing production of protective IFNg by *Mtb*-specific CD4^+^ T cells in pulmonary compartment of *Mtb*/SIV co-infected RMs [29]. In contrast to IFNg, cART + 3HP treatment resulted in a significantly lower percentage of *Mtb*-specific CD4^+^ T cells to produce TNFa in response to stimulation with either ESAT-6/CFP-10 (Fig. 4C) (*P = 0.03*) or *Mtb* CW (Fig. 4D) (*P = 0.009*) compared to cART treated RMs. It has been reported previously that T-cell derived TNFa is essential for sustained protection during chronic *Mtb* infection [30] and that TNFa can promote proliferation of effector T cells resulting in increased immunogenicity [31, 32]. It has been demonstrated that antigen-specific expression of TNFa in the absence of IFNg on CD4^+^ T cells in *Mtb*-infected patients strongly correlates with the potential to develop active TB, while the opposite phenotype is supportive of latent infection [33, 34]. Our results therefore suggest that concurrent cART + 3HP treatment results in the clearance of bacterial infection. Thus, concurrent treatment with cART + 3HP does not result in increased production of *Mtb*-specific TNFa which in turn has a detrimental impact on effector function needed for sustained protection. Similar to IFNg, a significant increase in IL-17^+^CD4^+^ T cells was observed in BAL of cART + 3HP treated RMs when stimulated with ESAT-6/CFP-10 (Fig. 4E) (*P = 0.01*) and *Mtb* CW (Fig. 4F) (*P = 0.005*) compared to cART treated RMs. The trends were similar in lung with significantly higher percentage of CD4^+^T cells expressing IFNg (*P = 0.04*) and IL-17 (*P = 0.01*) when stimulated with ESAT-6/CFP-10 (Fig. 4G) or *Mtb* CW (Fig. 4H) compared to cART treated RMs. While the role of T_H1_ cells is clearly associated with protection in *Mtb* infection through IFNg production, the role of T_H17_ cells is complex and is associated with tissue damage on one hand and anti-inflammatory response on the other hand. However, our findings align with the recent studies that show that *Mtb*-responsive IL-17-producing CD4^+^ T cells are preserved in humans with LTBI with HIV-ART and that IL-17 producing CD4^+^ T cells constitute the dominant response to *Mtb* antigen [35]. Moreover, we did not observe an increase in levels of pro-inflammatory cytokines, IL-6 and IP-10 in cART + 3HP treated RMs compared to cART treated RMs (Fig. 4I). Overall, there is an increased T_H1_/T_H17_ *Mtb*-specific response in cART + 3HP treated RMs that associates with protection but also has the potential to be pathological. In contrast we observed a decreased *Mtb*-specific TNFa response after concurrent treatment that could have detrimental impact on long term protection.

To better understand immune responses after concurrent cART + 3HP treatment relative to cART-treatment, we assessed transcriptional profiles of lung cells collected at necropsy from *Mtb*/SIV co-infected, cART or cART + 3HP treated RMs by RNA sequencing (Fig. 4J). *Mtb* is known to manipulate cell death pathways to evade host immunity, thereby protecting the bacilli from antibiotics, and allowing dissemination when timing is appropriate [36].Gene terms associated with cell death, apoptosis, death receptor signaling, and necrosis were highly enriched amongst induced genes from the lungs of cART + 3HP treated, compared to cART treated RMs (Fig. 4J). The increased expression of apoptosis-related genes could also be attributed to presence of antibiotics (isoniazid and rifapentine) that are known to cause oxidative damage in host cells, leading to increased apoptosis in addition to *Mtb* control [37]. An increased expression of Type I IFN genes, such as IFNA2, IFNA1/IFNA13 was seen in cART + 3HP treated RMs compared to cART treated RMs (Fig. 4J). The role of Type I IFN in TB is ambiguous. Both human and animal studies show evidence for the role of Type I IFN in *Mtb* expansion and disease pathogenesis [38]. Murine data particularly suggests that Type I IFN signaling promotes TB progression. Our own data from RMs suggests that pDC expressing Type I IFN associate with TB progression [39, 40]. A human blood transcriptional signature also largely comprised of Type I IFN response genes was described in TB patients [41] and validated in macaques with TB [42]. We have previously shown the enrichment of the Type I IFN signatures among the lymphoid cell clusters from the lungs of *Mtb*-infected mice [43]. Together, these results suggest a pathological role for Type I IFN in TB. Thus, our finding of an increased Type I IFN signature aligns with previously reported transcriptional signatures in human and NHP experiments [41, 44] and suggests that while clinical disease is controlled by concurrent therapy, these animals continue to harbor molecular signatures associated with TB pathology and immune activation in the lung.

### Single cell transcriptomic signature in pulmonary compartment of Mtb/SIV co-infected RMs.

**Figure 5:**
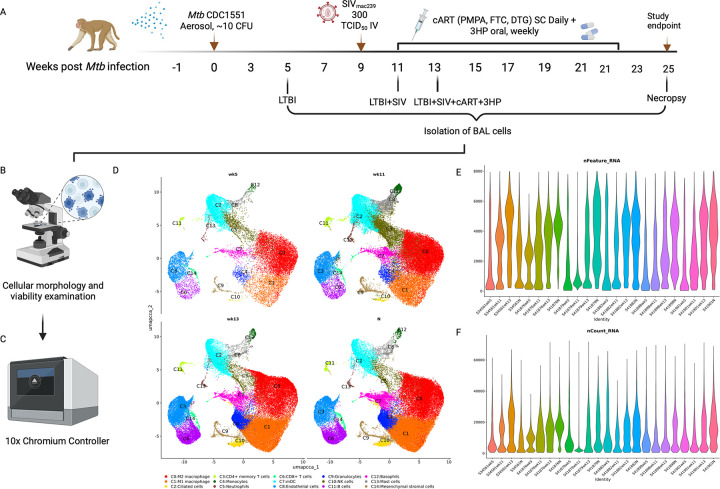
*Single cell transcriptomic signature in pulmonary compartment of Mtb/SIV co-infected RMs*. (A) Study outline for BAL sample collection from *Mtb*/SIV co-infected RMs treated with 12-weeks, once oral, cART+3HP (*n* = 6). (B) Quality control of BAL cells in terms of percent viability using automated cell countess, manual counts using trypan blue and microscopic evaluation. (C) BAL cells are then loaded onto Chromium controller (10x Genomics) to generate single-cell beads in emulsion. (D) Uniformed Manifold Approximation and Projection (UMAP) clustering to identify transcriptionally distinct clusters at weeks 5, 11, 13 and necropsy. (E) The total number of transcripts detected within each cell at weeks 5, 11, 13 and necropsy. (F) The total number of molecules detected within each cell at weeks 5, 11, 13 and necropsy.

We further investigated the transcriptional changes at single cell level in the pulmonary compartment of *Mtb*/SIV co-infected RMs treated with cART + 3HP. We collected BAL at four critical time points from the same RMs during the study period; week 5 (represents the asymptomatic phase of *Mtb* infection), week 11 (represents 2 weeks post-SIV co-infection), week 13 (represents post-SIV co-infection and 4 weeks of cART treatment) and necropsy (study endpoint after 12 weeks of cART + 3HP treatment) (Fig. 5A; Supplementary Fig. 6A, 6B). Using this experimental design, we were able to track the early transcriptomic changes in defined populations of cells at four different stages of *Mtb*/SIV co-infection. This negates the need for LTBI- and cART-naïve controls since they are represented by week 5 and week 11 timepoints in this study. All samples passed quality control in terms of cell quality (fraction reads in cells) and sequencing after which they were run on 10x chromium controller (Supplementary Table 1; Fig. 5B and 5C). Uniformed Manifold Approximation and Projection (UMAP) clustering identified 14 transcriptionally distinct cell clusters across all samples that can be broadly classified into lymphoid, myeloid and non-lymphoid, non-myeloid (Fig. 5D, Supplementary Fig. 7A, 7B). Lymphoid clusters include C3 (CD4^+^ memory T cells; *ADAM23*^*+*^, *CAMK4*^*+*^, *CD96*^*+*^, *CLEC2D*^*+*^, *ITK*^*+*^), C6 (CD8 T cells; *CCR5*^*+*^, *CD3D*^*+*^, *CD3E*^*+*^, *CD8A*^*+*^, *CD8B*^*+*^, *ITM2A*^*+*^, C10 (NK cells; *NCAM1*^*+*^, *EOMES*^*+*^, *GNLY*^*+*^, *GZMA+, KLRB1*^*+*^, *HOPX*^*+*^), C11 (B cells; *AFF3*^*+*^, *AKAP2*^*+*^, *BLK*^*+*^, *CD19*^*+*^, *CD79A*^*+*^, *CNR2*^*+*^, *CR2*^*+*^, *EBF1*^*+*^); Myeloid clusters include C0 (M2 macrophages; *MRC1*^*+*^, *ALDH2*^*+*^, *APOE*^*+*^, *ARL11*^*+*^, *CD63*^*+*^, *CD14*^*+*^, *GSTO1*^*+*^, *RAB13*^*+*^, *DNASE2B*^*+*^), C1 (M1 macrophages; *IL-6+, IL-8+, SLC11A1*^*+*^), C4 (Monocytes; *CD14*^*+*^, *CD163*^*+*^, *CD68*^*+*^), C5 (Neutrophils; *ABHD2*^*+*^, *ANO2*^*+*^, *CACNA1D*^*+*^, *CACNB4*^*+*^, *HAL*^*+*^, *MCTP1*^*+*^, *MITF*^*+*^, *TCF7L2*^*+*^), C7 (mDC; *CD1A*^*+*^, *CLIC2*^*+*^, *DSE*^*+*^, *FLT3*^*+*^, *EMP1*^*+*^, *P2RY6*^*+*^), C9 (Granulocytes; *FCGR3*^*+*^, *FPR1*^*+*^, *MNDA*^*+*^, *CSF3R*^*+*^), C12 (Basophils; *CD63*^*+*^, *ENPP3*^*+*^), and C13 (Mast cells; *CD117*^*+*^, *CD203c*^*+*^, *CD63*^*+*^). Non lymphoid non myeloid clusters include C2 (Ciliated cells; *STK11*^*+*^, *MARK3*^*+*^), C8 (Endothelial cells; *FOXJ1*^*+*^, *DNAH5*^*+*^, *TEKT1*^*+*^) and C14 (mesenchymal stromal cells; *CD44*^*+*^, *CD79A*^*+*^).

**Figure 6:**
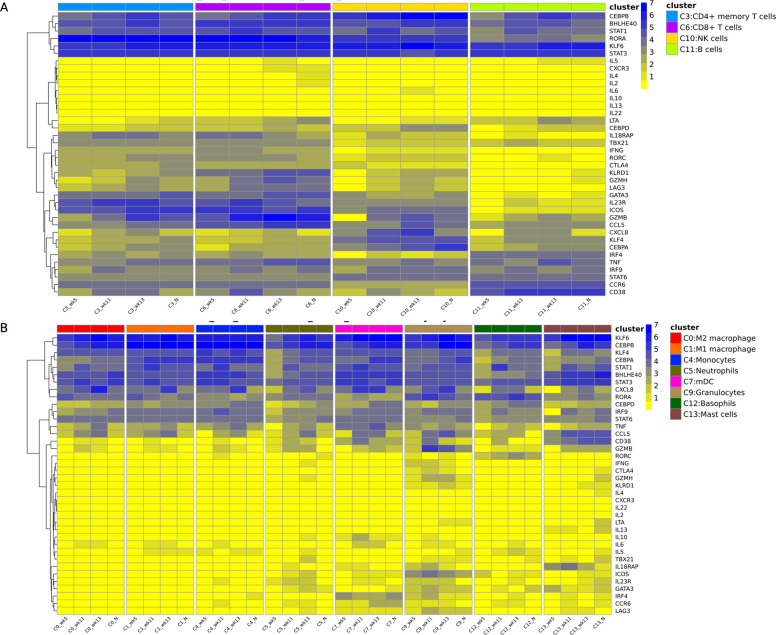
*T*_*H1*_*/T*_*H17*_ *signature in cART+3HP treated RMs*. Heatmap analysis of genes associated with T_H1_, T_H2_ and T_H17_ response in (A) lymphoid clusters and (B) myeloid clusters from BAL of *Mtb*/SIV co-infected RMs treated with cART+3HP at weeks 5, 11, 13 and necropsy (n = 6).

The total number of transcripts (nFeature_RNA) and molecules (nCount_RNA) detected within each cell increased in early phase of SIV co-infection compared to LTBI phase (Figs. 5E and 5F). Cells were filtered to detect genes within the range of 10–8000 to remove extremely low and high counts. The plot shows the distribution of detected gene levels of cells, and the colored shapes represent the distribution density (Figs. 5E and 5F). The nFeature_RNA and nCount_RNA remained at higher levels at the end of cART + 3HP treatment (necropsy time point) compared to LTBI phase of study (wk 5 time point). Based on published signature gene list, we analyzed T_H1_ (*TBX21, IFNG, TNF, LTA, IL18RAP, BHLHE40, STAT1*), T_H2_ (*IL-4, IL-5, IL-6, IL-10, IL-13, KLF4, TCR*) and T_H17_ (*CCR6, RORA, RORC, IRF4, STAT3, IL23R, IL22*) associated transcriptional changes in lymphoid (Fig. 6A) and myeloid (Fig. 6B) clusters at the pre-determined time points in BAL of *Mtb*/SIV co-infected, cART + 3HP treated RMs (Supplementary Fig. 8). Relative to the LTBI phase time point (wk 5), an increased expression of genes *BHLHE40, STAT1, RORA, STAT3, KLF6* was observed in lymphoid clusters and myeloid clusters at end of treatment with cART + 3HP (Fig. 6A, 6B and Supplementary Fig. 9). IL23R was expressed at higher levels at all time points in CD4^+^ memory T cell and CD8^+^ T cell clusters. CD8^+^ T cell cluster showed increased expression of activation marker genes; *KLRD1, CCL5, GZMB, GZMH, CTLA4, ICOS, LAG3*. However, it is to be noted that not all T_H1_ and T_H17_ associated genes were up regulated in lymphoid and myeloid clusters post cART + 3HP treatment. We did not observe an increased expression of *IL2, TBX21, IFNG, TNF, LTA, IL18RAP, IL22, RORC, IRF4, CCR6* at necropsy (end of cART + 3HP) compared to wk 5 post *Mtb* infection (LTBI phase) (Fig. 6A, 6B and Supplementary Fig. 9). Negligible expression of T_H2_-associated genes was observed at all time points in both lymphoid and myeloid clusters (Fig. 6A, 6B and Supplementary Fig. 9) except for high expression of KLF4 in myeloid clusters. Additionally, there was a high expression of LAG3, an exhaustion marker, and CD38, an immune activation marker in CD8^+^ T cell cluster post SIV co-infection at wk 11 and at end of cART + 3HP treatment at necropsy. Overall, we hypothesize that cART + 3HP mediates the increased T_H1_/T_H17_ response in pulmonary compartment through increased expression of *BHLHE40, STAT1, RORA* and *STAT3*.

## Discussion

We report here for the first time the impact of WHO-recommended cART + 3HP treatment regimen on LTBI reactivation in *Mtb*/SIV co-infected rhesus macaques in the presence of cART. As such, our results provide unprecedented, novel insights into the host response to co-infection and concurrent treatment. 3HP combines high dose isoniazid and rifapentine and is a once weekly, 12-week therapy taken orally. In humans, 3HP is associated with significantly lower hepatotoxicity and higher rates of completion than isoniazid preventive treatment [45, 46]. It is important to note that 3HP is a recommended regimen to treat LTBI and prevent TB in persons living with HIV. Recent clinical trials (Dolphin-study) have shown that for people starting anti-HIV treatment, combining dolutegravir containing cART with 3HP TB preventive treatment is safe and works efficiently in tandem [47] with high rates of viral suppression. Modeling concurrent cART + 3HP in *Mtb*/HIV co-infection using a relevant animal model, such as NHPs, provides an invaluable tool to investigate the impact on local immune responses. The NHP model is attractive for studying human *Mtb* infection and for performing preclinical studies on treatment regimens as it recapitulates key aspects of human *Mtb* infection states and TB disease [48–51]. Our group has previously shown that earlier initiation of cART suppresses the virus, partially reconstitutes CD4^+^ T cells but fails to control inflammation and immune activation [15, 20]. We have also shown that administration of 3HP failed to sterilize bacteria in the lung of latently infected RMs with 2 of the 6 RMs showing culturable *Mtb* in the lungs (~ 3 logs), 4 to 5 weeks post-SIV co-infection [13]. In this study, we sought to determine if concurrent cART + 3HP therapy initiated at early stages of co-infection better controls immune dysfunction in pulmonary compartment compared to cART.

Administration of concurrent cART + 3HP improved the clinical and microbiological attributes of *Mtb*/SIV co-infection compared to cART naïve or cART treated RMs. RMs were trained to take 3HP orally mimicking humans. As seen in the DOLPHIN study, our model demonstrated that co-administration of dolutegravir with 3HP was safe, well-tolerated and did not require any dose-adjustment of dolutegravir. Initiation of cART and 3HP at 2 weeks post *Mtb*/SIV co-infection sterilized bacterial burden in lung of 5 out of 6 RMs and completely prevented dissemination to extra-pulmonary organs in all 6 RMs. There was a significant reduction in percent lung involvement in pathology in cART + 3HP treated RMs with visibly fewer granulomas compared to cART naïve or cART-treated RMs. The few granulomas observed at end of cART + 3HP treatment were characterized as an equal mix of non-necrotizing and caseous type. 18F-FDG PET/CT scans revealed a significant reduction in number of lesions post treatment with cART + 3HP but not in uptake of 18F-FDG in the few lesions that remained at ned of treatment. Taken together, cART + 3HP treatment exerts bacterial and viral control, thereby improving the health status of *Mtb*/SIV co-infected RMs during the study period. However, cART + 3HP treated RMs continued to harbor granulomas that have the potential to release infectious bacilli and exhibit increased 18F-FDG uptake associated with inflammation.

We next investigated immune reconstitution in the pulmonary compartment of RMs treated with cART + 3HP compared to LTBI, cART naïve and cART-treated RMs. We have previously shown that cART is unable to reconstitute CD4^+^ T cells in the lung tissue to the levels seen in LTBI and that the reconstituted CD4^+^ T cells are dysfunctional for *Mtb*-specific response [15, 20]. Concurrent administration of cART and 3HP did not further improve the frequency of reconstituted CD4^+^ T cells in lung of *Mtb*/SIV co-infected RMs compared to cART only treated RMs. The reconstituted CD4^+^ T cells in BAL and lung of cART + 3HP treated RMs exhibited an increased frequency of activated and inflamed phenotype compared to LTBI RMs. Activated CD4^+^ T cell phenotype associates with high risk for TB progression. Our model therefore demonstrates that SIV-induced activation of pulmonary CD4^+^ T cells is not ameliorated by cART + 3HP. A majority of reconstituted CD4^+^ T cells appeared to be central memory phenotype. On the contrary, there was a significant reduction in the effector memory CD4^+^ T cells population in pulmonary compartment post SIV co-infection that cART + 3HP did not alleviate as was also seen in cART treated RMs. CD4^+^ T_EM_ cells are critical for host protection to subsequent antigen encounter. The effector memory CD4^+^ T cells can produce early effector cytokines such as IFNg and TNFa that help activate other cell types such as CD8^+^ T cells or they can directly kill the infected cells. It is feasible that reduced bacterial burden results in reduced antigen presentation which can cause a reduced frequency of CD4^+^ T_EM_ cell in cART + 3HP treated RMs. However, chronic *Mtb* infection such as a latent TB infection is known to elicit effector memory phenotype in CD4^+^ and CD8^+^ T cells [52]. Our model recapitulates this phenotype as is seen by > 10% CD4^+^ T_EM_ in BAL collected from the same RM during LTBI phase that reduces to less than 3% post SIV co-infection. Clearly, the presence of CD4^+^ T_EM_ associates with an immune balance seen in LTBI in our model and a decrease in the frequency of this cell type contributes to immune dysfunction that cART + 3HP fails to mitigate.

We next determined the percentage and functionality of *Mtb*-specific CD4^+^ T cells in pulmonary compartment of *Mtb*/SIV co-infected RMs treated with cART + 3HP compared to cART. We performed ex vivo stimulation of BAL cells isolated at week 5 (represents the asymptomatic phase of *Mtb* infection), week 11 (represents 2 weeks post-SIV co-infection), and necropsy (after 12 weeks of cART + 3HP treatment) with ESAT-6/CFP-10 and *Mtb* CW. Upon 12 weeks of cART + 3HP treatment, an increased percentage of IFNg and IL-17 producing *Mtb*-specific CD4^+^ T cells was seen in BAL and lung. Similar to what has been reported in humans, it is feasible that a majority of these T_H1_/T_H17_ cytokine producing cells in BAL and lung are of central memory phenotype since CD4^+^ T_CM_ were the dominant cell type observed in pulmonary compartment at end of cART + 3HP treatment [53]. On the contrary, a lesser percentage of TNFa- producing *Mtb*-specific CD4^+^ T cells was observed at the end of cART + 3HP treatment compared to cART treated RMs. TNFa is required for granuloma organization and inhibition of TNFa through TNFa inhibitors result in TB reactivation [54]. Hence, the skewed reconstitution of *Mtb*-specific response consisting of an increased IFNg and IL-17 response but a defective TNFa response could prove detrimental in long-term protection, altered granuloma formation and dissemination of disease.

Bulk RNA sequencing of lung tissue collected at necropsy from cART + 3HP treated RMs showed increased type I IFN response-associated genes; *“Interferon signaling”, “IFNA2”, “IFNA1/IFNA13”, “ifnar”, “interferon alpha”, “IRF9”, “IRF1”* and apoptosis genes; *“Apoptosis”, “Apoptosis of epithelial cells”, “cell death of progenitor cells”, “cell death of germ cells”, “Apoptosis of hematopoietic cells”* compared to cART treated RMs. Type I IFN are critical in host defense to viruses. However, there is a growing body of literature that describes the detrimental impact of type I IFN in *Mtb* infection [55, 56]. In humans, type I IFN is associated with loss of control and progression to TB disease [57, 58]. Recently, type I IFN was shown to play a role in *Mtb*-induced macrophage cell death that leads to release of bacilli from dead macrophages and dissemination. Previously, it was shown that the signaling pathways involved with type I IFN are involved in apoptosis [59, 60] that explains the concomitant increase in expression of genes associated with apoptosis in cART + 3HP treated RMs. Overall, RMs treated with cART + 3HP present a distinct transcriptomic signature that associates with immune cell death. A deeper analysis of immunological recovery at the single cell level confirmed increased expression of genes associated with immune control of *Mtb* including, CD4^+^ memory T cells, CD8^+^ T, NK cells, B cells, M1/M2 macrophages, granulocytes and epithelial cells. Concurrent with the flow cytometry data, scRNAseq showed an increased expression of certain T_H1_ and T_H17_-associated genes in lymphoid clusters at end of cART + 3HP treatment. CD8^+^ T cell cluster was characterized by an activated signature with substantially higher cytotoxic function-associated gene expression compared to CD4^+^ memory T cells, NK and B cells. One possibility could be that this increased cytotoxic gene signature in CD8^+^ T cell cluster associates with the increased apoptotic signature seen in bulk RNAseq since release of cytotoxic molecules by CD8^+^ T cells is known to cause apoptosis of target cells [61]. In humans on cART, increased expression of immune activation marker, CD38 on CD8^+^ T cells during chronic HIV infection associates with the inability to proliferate and increased exhaustion. Overall, it is important to note that while cART + 3HP effectively controls the virus and the bacilli, there is disproportionate reconstitution of memory subsets, levels of activation and exhaustion markers as well as their functional capacity.

There are some limitations to this study. Since functional restoration of CD4^+^ and CD8^+^ T cells is a gradual process in humans, our study, with a window of ~ 3 months post-treatment, may not recapitulate these settings exactly. We necropsied the RMs at the end of 12-week cART + 3HP treatment to match time points with previous cohorts. To study long-term immune reconstitution by cART + 3HP, we are now planning future studies with extended time to necropsy post treatment completion. Another caveat is that the model may not provide a full physiological recapitulation of human *Mtb*/HIV co-infection, because RMs are exposed to a supraphysiological dose of SIV. Not all humans on cART are likely to exhibit treatment failure and progression to TB reactivation. However, *Mtb*/HIV co-infected individuals on cART remain ~ 10-fold more likely to reactivate than HIV-naïve people with LTBI [62, 63]. Humans likely develop LTBI with a substantially lower infectious dose of *Mtb* (1–2 CFU) than we use to infect RMs (~ 10–15 CFU *Mtb* CDC1551). RMs infected with the CDC1551 dose/strain combination exhibit control of *Mtb* infection akin to human LTBI, yet the dose is higher than the physiologically relevant human infectious dose. Hence, our results are indicative of the worst outcomes in co-infected humans. We infect the RMs through aerosol, the natural route of infection, mimicking humans. *Mtb* strain, CDC1551 allows for the development of a human TB model resulting in a latent to chronic rather than active TB disease [48]. CDC1551 has also been shown to induce a protective immune response despite being similar in virulence to other lab strains [64]. Thus, our model allows for an in-depth analysis of the clinical and immunological response in the lung to cART + 3HP, which is possible only in a handful of research institutions world-wide. We are currently however, engaged in performing experiments with samples from human cohorts to validate our results.

In conclusion, while concurrent cART and 3HP effectively suppress the virus and bacteria, the quality of immune reconstitution in the pulmonary compartment remains significantly sub-optimal. cART + 3HP treatment increases the T_H1_/T_H17_ response in lung but there is incomplete restoration of protective, CD4^+^ T_EM_ and replenished *Mtb*-specific CD4^+^ T cells are skewed in their ability to produce TNFa. Though concurrent therapy improves pathological burden, there is increased 18F-FDG uptake in the few lesions that remain despite treatment. Further, transcript analysis of lung and BAL showed an increased expression of CD38, an immune activation marker on CD8^+^ T cells, as well as of apoptotic signature characteristic of cell death. Our results clearly show that despite the mitigation of co-infection, chronic immune activation persists in the lungs of concurrently treated NHPs. Targeting the host immune response via a host-directed immunotherapy provides an opportunity to augment immunity during the short-window of acute HIV-1 co-infection of *Mtb*. Future studies should perform testing of safety and efficacy of novel host-directed therapies such as IL-21-IgFc fusion protein administration or use of IDO-1 inhibitors concurrent to standardized therapies in tissues and organs like the lung, that are impossible to access in humans. This is critical for the development of an immune-based intervention along with cART and anti-TB therapy to control dysregulated immune responses generated during early events of HIV co-infection of LTBI and provide long-term immune reconstitution.

## Methods

### Animal infection.

This study included macaque data from completed studies [15, 19, 65]. A total of 18 specific pathogen free Indian-origin rhesus macaques (*Macaca mulatta*) were infected with a low dose of approximately 10 CFU M. *tuberculosis* CDC1551 (BEI Resources, catalog NR13649) via aerosol as described before [28, 66–68] (Supplementary Table 2). TST was performed at weeks 3 and 5 post TB infection to confirm infection. All the RMs were monitored for CRP, percent body weight and body temperature weekly through the study period. 14 of the LTBI RMs were then co-infected with 300 TCID_50_ SIVmac_239_ via the intravenous route 9 weeks post-TB infection [15, 17, 19, 65] (provided by the Preston Marx Laboratory, TNPRC, Covington, Louisiana, USA). All the procedures were conducted a board-certified veterinary clinician. The remaining 4 RMs served as LTBI controls for the study. The viral infection was confirmed through plasma viral loads via reverse transcription quantitative PCR (RT-qPCR). Upon confirmation of SIV infection, the 18 RMs were then divided into 3 groups: the first group of 8 RMs served as co-infected controls with no cART administration; the second group of 4 RMs were started on cART at 2 weeks post-SIV co-infection or 11 weeks post TB infection (cART at peak viremia) and the third group of 6 RMs started cART + 3HP at 2 weeks post-SIV co-infection once weekly for 12 weeks. All the RMs in cART-naive group had to be euthanized within 2–4 weeks of cART treatment due to clinical signs of TB reactivation. The RMs in the cART group were euthanized after 9 weeks of cART treatment while the RMs in cART + 3HP group were euthanized at end of 12-week treatment at week 24.

### *cART + 3HP* regimen.

Co-infected RMs received a drug regimen consisting of 20 mg/kg of (R)-9-(2-phosphonylmethoxypropyl) adenine (PMPA, tenofovir, Gilead Sciences), 30 mg/kg of 2’, 3’-dideoxy-5-fluoro-3’-thiacytidine (FTC, emtricitabine, Gilead Sciences) and 2.5 mg/mL of the integrase inhibitor, DTG, Dolutegravir (ViiV Healthcare). The drugs were administered daily via subcutaneous injection of a cocktail of these three drugs in the vehicle kleptose at previously published doses [19]. Co-infected RMs also received a weekly oral dose of 15mg/kg isoniazid and 15 mg/kg rifapentine for 12 weeks beginning week 12 after aerosol infection up to week 23 post-TB infection. Oral intake was monitored by veterinary staff to ensure consumption.

### Positron emission tomography-computed tomography (PET/CT) imaging.

Longitudinal CT and PET/CT scans were performed using MEDISO’s LFER150 PET-CT scanner at 3–6 week intervals, starting from week 6 post-*Mtb* infection with the last scan prior to necropsy [69]. Briefly, we performed 18F-fluorodeoxyglucose (FDG) PET/CT scans for each anesthetized RM using the breath-hold technique. RMs were anesthetized and intubated under supervision of a board-certified veterinarian as per approved IACUC protocols. All the RMs received an intravenous injection of 1 mCi per kg of body weight dose of 18F-FDG [70], procured from Cardinal Health radiopharmacy. The single field of view (FOV) and/or double FOV lung CT scans were performed using breath-hold as described [71]. PET scans were acquired after completion of the 40–50 min FDG uptake period. Images were visualized using Interview Fusion 3.03 (Mediso) and reconstructed using Nucline NanoScan LFER 1.07 (Mediso) with parameters as described [72]. The lung segmentation, volumetric and SUV analysis was performed using Vivoquant 4.0 (Invicro, USA) [69].

### Viral load and bacterial burden measurement.

Bacterial burden in BAL was measured throughout the study period as previously described [17]. Viable *Mtb* burden was also measured at necropsy in BAL, lung, spleen, bronchial lymph node and individual granulomas collected at necropsy [17, 65]. Viral loads in acellular BAL supernatant and plasma were determined by RT-qPCR at peak viremia (2 weeks post-SIV or 11 weeks post TB-infection), week 13, week 15 post-*Mtb* infection and at necropsy. The measurements were performed by NIAID, DAIDS, Nonhuman Primate Core Virology Laboratory for AIDS Vaccine Research and Development). A lower limit of 100 copies/ sample was set for quantification of SIV copies in this assay.

### High parameter flow cytometry.

High parameter flow cytometry was performed on BAL cells at pre-infection, pre-SIV (wk 3, 5), post-SIV, pre-cART (wk 11), post-cART (wk 20 or necropsy) and post-cART + 3HP (wk 24 or necropsy). Lung, bronchial lymph nodes and granulomas were harvested at necropsy and processed as described earlier [15, 17, 65]. The single cells prepared were then stained with surface and intracellular markers to study various cell phenotypes (Supplementary Table 3). The freshly collected BAL cells were stimulated *ex vivo* with *Mtb*-specific antigens, ESAT-6/CFP-10 and *Mtb* Cell Wall Fraction (BEI Resources, 10 μg/mL) for a total of 16 h. Brefeldin A (0.5 μg/mL, SIGMA) was added 2 h after the onset of stimulation. After stimulation, the cells were stained with LIVE/DEAD fixable Near-IR stain (ThermoFisher) and stained subsequently with the surface antibodies: CD4-PerCP-Cy5.5 (BD Biosciences), CD8-APC (BD Biosciences), CD3-AlexaFlour 700 (BD Biosciences), CD95-BV421 (BD Biosciences), CD28-PECy7 (BD Biosciences) and CD45-BUV395 (BD Biosciences). Cells were then fixed, permeabilized and stained with intracellular antibodies: IFNγ - APC-Cy7 (Biolegend), IL-17-BV605 (Biolegend) and TNFα - BV650 (Biolegend). Cells were washed, suspended in BD stabilizing fixative buffer and acquired on BD FACS Symphony flow cytometer. Analysis was performed using FlowJo (v10.6.1) using previously published gating strategy [15, 17, 19, 68].

### Gross pathology.

The animals were euthanized for necropsy and lung lobes, spleen, liver, bronchial lymph nodes were collected. All the tissues were weighed at the time of collection. Tissues were fixed in 10% neutral-buffered formalin, paraffin embedded, sectioned at 5 μm thickness and stained with hematoxylin and eosin using standard methods. Lung tissues were collected stereologically at necropsy and stereology scores were prepared on percentage lung affected by a board-certified veterinary pathologist.

### Immunohistochemistry staining.

Fluorescent immunohistochemistry was performed on formalin-fixed, paraffin-embedded lung and bronchial lymph node tissues as previously described [15, 16, 19, 65, 73]. The stained slides were scanned in the Axio Scan Z1 and the images were analyzed using HALO software.

#### Study Approval

All infected animals were housed under Animal Biosafety Level 3 facilities at the Southwest National Primate Research Center, where they were treated according to the standards recommended by AAALAC International and the NIH guide for the Care and Use of Laboratory Animals. The study procedures were approved by the Animal Care and Use Committee of the Texas Biomedical Research Institute.

### Quality control for frozen BAL cells.

Prior to running the BAL cells on 10x Genomics platform, the cells were analyzed for viability using i) automated cell countess, ii) manual counts using Trypan Blue and iii) microscopic evaluation. Briefly, cells were thawed on ice. 100 μL of cells was washed once in 1 mL warmed 1x phosphate buffered saline (PBS) (Gibco), centrifuged, and resuspended in 1 mL of 1x PBS. Cells were mixed in 1:1 ratio with Trypan blue and counted in automated countess as well by hemocytometer (Supplementary Table 1). Cellular morphology, including shape and size was determined using a standard bright field light microscope. Institutional approved protocols were applied when removing samples from BSL3.

### Single cell RNA Library generation and sequencing.

BAL cell suspensions were loaded onto Chromium instrument (10x Genomics) to generate single-cell beads in emulsion. Single-cell RNA-seq libraries were then prepared using Single Cell 3’ Gel bead and library kit version 3.1 (10× Genomics). Single cell barcoded cDNA libraries were quantified and sequenced on an Illumina NovaSeq 6000. Read lengths were 28bd for read 1, 10bp for index 1, 10bp for index 2, and 100bp for read 2. Cells were sequenced to about 50,000 reads per cell.

### Single cell data analysis.

Cell ranger Single Cell Software suite (V7.0.1) from 10x was used to perform sample demultiplexing and generate fastq files. Resulting fastq files were aligned against reference genome mmul10 (Genebank, https://www.ncbi.nlm.nih.gov/datasets/genome/GCF_003339765.1/) with cellranger count. The targeted cell recovery per sample was set to 10,000 cells. The cellranger counting results for 16 samples were further integrated and analyzed by R software with package Seurat (V4.4.0). The data matrix for each sample was read by Read10X and filtered by removing cells which have more than 8000 detected genes in each sample. All 16 samples data were merged, normalized with method “LogNormalize”, and most variable genes were detected by the FindVariableFeatures function with nfeatures 2000. Anchor genes were selected by SelectIntegrationFeatures and FindIntegrationAnchors, and further applied to integrated dataset by IntegrateData. The integrated data were scaled by ScaleData and principal component analysis [74] was performed by RunPCA with npcs = 30. To visualize the data, the TSNE dimensionality reduction was performed using the first 20 PCA. Data clustering was run by FindNeighbors (pca 20) and FindClusters (resolution 0.2). Basic marker genes for each cluster were firstly identified using FindAllMarkers function in Seurat R package by (logFC.threshold > 0.25, minPct > 0.1), then the marker genes with different cut-off were further studied and evaluated. Heatmaps were created by Seurat Package using the mean expression of markers in each cluster per time point.

## Data Availability

The single cell RNAseq raw and processed files are available at NCBI Gene Expression Omnibus and the accession number is xxxxx.
